# Exploratory Analysis of the Effects of Thymoquinone Alone and in Combination with 5-Fluorouracil on Inflammatory Mediators in Colorectal Cancer Cells: An In Vitro and In Silico Study

**DOI:** 10.3390/molecules31193415

**Published:** 2026-09-25

**Authors:** Natalia Kurowska, Marcel Madej, Maria Książek, Barbara Strzałka-Mrozik

**Affiliations:** 1 Department of Molecular Biology, Faculty of Pharmaceutical Sciences in Sosnowiec, Medical University of Silesia, 41-200 Sosnowiec, Poland; d201167@365.sum.edu.pl (N.K.); maria.ksiazek@sum.edu.pl (M.K.); 2 Silesia LabMed, Centre for Research and Implementation, Medical University of Silesia, 40-752 Katowice, Poland

**Keywords:** colorectal cancer, thymoquinone, 5-fluorouracil, COX-2, IL-6, IL-8, inflammatory mediators, molecular docking

## Abstract

Colorectal cancer (CRC) remains one of the leading causes of cancer-related morbidity and mortality worldwide. Chronic inflammation plays a pivotal role in CRC development and progression, with cyclooxygenase-2 (COX-2), interleukin-6 (IL-6), and interleukin-8 (IL-8) representing key mediators of tumor-promoting inflammation. This study aimed to evaluate the effects of thymoquinone (TQ), 5-fluorouracil (5-FU), and their combination on the expression of COX-2, IL-6, and IL-8 at both the mRNA and protein levels in colorectal cancer cell lines (SW1116 and RKO) and normal colon epithelial cells (CCD-841CoN). Although numerical changes in inflammation-associated mediator expression were observed following TQ monotherapy, none of the TQ-monotherapy comparisons versus the untreated control reached statistical significance after correction for multiple comparisons. Notably, combined TQ/5-FU treatment significantly reduced COX-2 protein levels in SW1116 cells compared with the untreated control. Differences between transcript and protein responses were observed across the investigated cell lines and treatment conditions. However, since both endpoints were assessed at a single time point, the mechanisms underlying these differences cannot be conclusively determined from the present data. Exploratory molecular docking analysis was additionally performed to examine potential direct interactions of TQ with COX-2, IL-6, and IL-8. The predicted docking scores ranged from −5.1 to −6.6 kcal/mol, with the most favorable predicted interaction observed for COX-2. These computational findings are hypothesis-generating and should be interpreted independently of the experimentally observed changes in target expression. Collectively, the present findings demonstrate treatment- and cell-line-dependent response patterns in inflammation-associated mediators, while providing no statistically significant evidence of an effect of TQ monotherapy versus the untreated control under the present experimental conditions.

## 1. Introduction

Colorectal cancer (CRC) is the third most commonly diagnosed malignancy and the second leading cause of cancer-related mortality worldwide, representing a major global health burden [1,2]. Despite substantial advances in prevention, diagnosis, and treatment, the incidence of CRC continues to increase, particularly among younger individuals. More than half of CRC cases are attributable to modifiable lifestyle-related risk factors, including tobacco smoking, unhealthy dietary habits, excessive alcohol consumption, physical inactivity, and overweight or obesity, highlighting the considerable potential for disease prevention [1]. Of particular concern is the rising incidence of early-onset CRC. Recent estimates indicate that nearly 45% of newly diagnosed CRC cases occur in individuals younger than 65 years, compared with only 27% in 1995. In the United States alone, approximately 55,230 CRC-related deaths are projected in 2026, with almost one-third occurring in patients under the age of 65 [1].

Recent research has increasingly highlighted the role of environmental and lifestyle factors in colorectal carcinogenesis through their effects on the intestinal microenvironment and chronic inflammation. Particular attention has been given to gut microbiota dysbiosis and inflammatory responses associated with exposure to ultra-processed foods, microplastics, and widespread antibiotic use, all of which have been implicated in CRC development [1]. Chronic inflammation is now recognized as a key driver of CRC initiation and progression. Patients with inflammatory bowel disease (IBD) are at a significantly increased risk of developing CRC. A meta-analysis by Lutgens et al. reported a 1.7-fold higher relative risk of CRC in patients with IBD compared with the general population [3]. Likewise, ulcerative colitis (UC) is associated with an approximately 2.4-fold increased risk of CRC (95% CI: 2.1–2.7) after 14 years of follow-up, with nearly 1.6% of patients developing colorectal cancer. The risk is even greater in patients with extensive colitis, reaching a 5.7-fold increase, while male patients with UC have a higher CRC risk than female patients (2.6-fold versus 1.9-fold, respectively) [2]. These observations underscore the central role of chronic inflammation in colorectal carcinogenesis and provide the rationale for targeting inflammation-associated signaling pathways as a therapeutic strategy.

The tumor-promoting effects of chronic inflammation are mediated through a complex network of cytokines, chemokines, and inflammatory enzymes that collectively regulate tumor initiation, progression, metastasis, and resistance to therapy. Persistent inflammatory signaling reshapes the tumor microenvironment (TME) through the coordinated activity of immune cells and pro-inflammatory mediators, including tumor necrosis factor-α (TNF-α), interleukin-1β (IL-1β), and interleukin-6 (IL-6), thereby promoting cancer cell survival, proliferation, angiogenesis, and immune evasion [4]. Recent evidence indicates that the dynamic interactions within the TME are critical for the adenoma-to-carcinoma transition in colorectal cancer. Rather than acting independently, inflammatory mediators form a highly interconnected signaling network. IL-1β is one of the major upstream regulators, inducing the expression of IL-6, interleukin-8 (IL-8), and cyclooxygenase-2 (COX-2), thereby amplifying the inflammatory response and establishing a positive feedback loop that sustains tumor-promoting inflammation. Activation of the nuclear factor kappa B (NF-κB) pathway further reinforces this network by maintaining elevated expression of IL-6 and IL-8, two cytokines that promote cancer cell proliferation, resistance to apoptosis, angiogenesis, and metastatic potential [5]. Notably, increased IL-8 mRNA expression has been reported throughout the adenoma-to-carcinoma sequence, suggesting that dysregulation of this signaling pathway occurs early during colorectal tumorigenesis [6].

Another key component of this inflammatory network is cyclooxygenase-2, whose expression is markedly elevated in colorectal adenocarcinomas compared with adjacent normal mucosa. Increased COX-2 expression has also been detected during the adenoma-to-carcinoma sequence, underscoring its role in both tumor initiation and progression [6]. Through the synthesis of prostaglandin E2 (PGE2), COX-2 promotes cancer cell proliferation, angiogenesis, invasion, and metastatic dissemination to distant organs, including the liver, lymph nodes, bone, and brain [7]. Accumulating evidence further demonstrates extensive crosstalk among the IL-6, IL-8, and COX-2 signaling pathways, which sustains a chronic pro-inflammatory tumor microenvironment and promotes colorectal cancer progression. Consequently, the IL-6/IL-8/COX-2 inflammatory network represents a potentially relevant target for therapeutic modulation in CRC.

Despite substantial advances in colorectal cancer management, treatment efficacy remains limited by drug resistance, adverse effects, and disease recurrence. Fluoropyrimidine-based chemotherapy, particularly 5-fluorouracil (5-FU), remains the cornerstone of systemic treatment for CRC. However, its clinical efficacy is frequently compromised by intrinsic and acquired resistance mechanisms, resulting in limited and often transient therapeutic responses [8,9,10]. Moreover, resistance to 5-FU may reduce sensitivity to other cytotoxic agents, highlighting the need for complementary therapeutic strategies capable of targeting tumor-associated molecular pathways and improving treatment outcomes. Among the signaling pathways involved in the regulation of this inflammatory network, NF-κB represents an important molecular hub linking inflammatory signaling with colorectal cancer progression.

Increasing evidence suggests that therapeutic strategies targeting multiple aspects of cancer biology, including inflammatory signaling, may complement conventional anticancer therapies. Consequently, compounds capable of modulating inflammation-associated signaling pathways have attracted considerable attention as potential adjuvant agents in cancer treatment. Thymoquinone (TQ), the major bioactive constituent of *Nigella sativa*, has emerged as a promising natural compound with diverse biological activities, including anticancer, anti-inflammatory, and immunomodulatory effects [11,12,13]. Previous studies have demonstrated that TQ modulates multiple signaling pathways involved in cancer progression, particularly NF-κB-mediated inflammatory signaling. In addition, TQ has been reported to downregulate the expression of several pro-inflammatory mediators and tumor-promoting factors, including IL-1β, TNF-α, COX-2, PGE2, and matrix metalloproteinases, thereby limiting inflammation-driven tumor progression [11,14,15]. Furthermore, TQ has been reported to enhance natural killer cell activity, suggesting an additional potential role in antitumor immune responses [15].

Although the anticancer and inflammation-modulating properties of TQ have been investigated in several colorectal cancer models, its effects on the IL-6/IL-8/COX-2 inflammatory axis, particularly in combination with 5-FU, remain incompletely characterized. Therefore, the present study investigated the effects of TQ, alone and in combination with 5-FU, on the expression of IL-6, IL-8, and COX-2 at both the mRNA and protein levels in colorectal cancer cell lines and normal colon epithelial cells. In addition, exploratory molecular docking analysis was performed to examine the predicted interactions of TQ and 5-FU with these inflammation-associated proteins. The docking analysis was considered independently of the experimental expression data and was not intended to establish a causal relationship between predicted ligand–protein interactions and changes in target expression.

## 2. Results

The effects of TQ, 5-FU, and their combination on the expression of COX-2, IL-6, and IL-8 were evaluated at both the mRNA and protein levels in colorectal cancer cell lines (RKO and SW1116) and normal colon epithelial cells (CCD-841CoN). In addition, molecular docking analysis was performed to investigate the predicted interactions of TQ and 5-FU with these inflammatory targets.

### 2.1. Effect of TQ and 5-FU on COX-2 Expression

Treatment-induced changes in COX-2 expression are shown in Figure 1. At the mRNA level, no statistically significant differences were observed among the treatment groups in CCD-841CoN or SW1116 cells (Figure 1A,C). In RKO cells, the TQ/5-FU combination significantly increased *PTGS2* mRNA expression compared with the untreated control (*p* = 0.0395; Figure 1E), whereas no other comparisons reached statistical significance.

At the protein level, the TQ/5-FU combination significantly reduced COX-2 levels compared with the untreated control in SW1116 cells (*p* = 0.0279; Figure 1D). No other comparisons reached statistical significance. No statistically significant differences in COX-2 protein levels were detected among the treatment groups in CCD-841CoN or RKO cells (Figure 1B,F).

### 2.2. Effect of TQ and 5-FU on IL-8 Expression

Treatment-induced changes in IL-8 expression are shown in Figure 2. At the mRNA level, treatment with 5-FU significantly increased *CXCL8* transcript levels in RKO cells compared with the untreated control (*p* = 0.0194; Figure 2E). No other statistically significant differences in *CXCL8* mRNA expression were observed among the treatment groups.

At the protein level, cell-line-dependent differences were observed. In RKO cells, IL-8 protein levels were significantly higher following 5-FU treatment than following treatment with the TQ/5-FU combination (*p* = 0.0134; Figure 2F). In CCD-841CoN cells, a significant difference in IL-8 protein levels was observed between the TQ and TQ/5-FU groups (*p* = 0.0279; Figure 2B). No other statistically significant differences in IL-8 protein levels were detected among the treatment groups (Figure 2A,C,D).

### 2.3. Effect of TQ and 5-FU on IL-6 Expression

Treatment-induced changes in IL-6 expression are shown in Figure 3. At the mRNA level, no significant differences were observed among the treatment groups in any of the three cell lines (Figure 3A,C,E).

At the protein level, cell-line-dependent differences were observed. In SW1116 cells, TQ treatment resulted in a numerical decrease in IL-6 protein levels compared with the untreated control; however, this difference did not reach statistical significance (*p* = 0.07; Figure 3D). In CCD-841CoN cells, 5-FU treatment significantly decreased IL-6 protein levels compared with the untreated control (*p* = 0.0134; Figure 3B). No statistically significant differences in IL-6 protein levels were detected among the treatment groups in RKO cells (Figure 3F).

### 2.4. Molecular Docking Analysis of TQ and 5-FU Interactions with Inflammatory Mediators

To investigate the potential interactions of TQ and 5-FU with the selected inflammatory mediators, molecular docking analysis was performed.

The predicted favorability of the protein–ligand interactions was assessed based on docking scores, with more negative values indicating more favorable predicted interactions. Detailed protein–ligand interactions analyzed in the present study are summarized in Table 1 and Table 2 and illustrated in Figure 4. The previously reported docking results for the 5-FU–IL-6 complex are presented in Table 2 and Figure 5A of Reference [16], whereas those for the 5-FU–IL-8 complex are reported in Tables 1 and 2 and Figure 9A of Reference [17]. These previously published data are therefore not discussed in detail here.

Molecular docking analysis showed that TQ had the most favorable predicted docking score for COX-2 among the analyzed inflammation-associated proteins, with a score of −6.6 kcal/mol (Table 1). TQ formed two hydrogen bonds with Tyr355 and Ser530. In addition, seven hydrophobic interactions and one π–alkyl interaction were identified within the predicted TQ–COX-2 binding pose.

TQ also formed a hydrogen bond with IL-8 (Val27), with a predicted docking score of −5.2 kcal/mol. A π–alkyl interaction was also identified. In contrast, the TQ–IL-6 complex showed a predicted docking score of −5.1 kcal/mol, with no hydrogen bonds identified. Additionally, an unfavorable bump interaction was observed for the TQ–IL-6 complex. Overall, these docking results should be interpreted as exploratory predictions of potential ligand–protein interactions rather than evidence of functional binding.

Celecoxib (CXB), a representative selective COX-2 inhibitor, was included as a reference compound in the docking analysis. CXB was docked into the validated COX-2 binding site using the same docking parameters as those applied to TQ. The best predicted docking score for CXB was −7.9 kcal/mol. The corresponding docking results are presented in Figure 5.

The predicted TQ binding pose was located within the cyclooxygenase channel of COX-2, with Val523 and Ser530 among the residues located within 5 Å of the ligand. This spatial arrangement supports the predicted localization of TQ within the COX-2 cyclooxygenase channel [18].

## 3. Discussion

Colorectal cancer remains one of the leading causes of cancer-related morbidity and mortality worldwide. Accumulating evidence indicates that chronic inflammation plays a central role in CRC initiation and progression, contributing to tumor growth, metastasis, and resistance to therapy [4,5]. Among the numerous inflammatory mediators implicated in colorectal carcinogenesis, COX-2, IL-6, and IL-8 are recognized as important components of the pro-inflammatory tumor microenvironment, promoting cancer cell proliferation, angiogenesis, invasion, metastasis, and immune evasion [4,5].

Although 5-fluorouracil-based chemotherapy remains a cornerstone of systemic treatment for CRC, its clinical efficacy is frequently limited by intrinsic and acquired resistance, as well as treatment-related toxicity [8,19]. Consequently, naturally occurring bioactive compounds have attracted considerable interest as potential adjuncts to conventional chemotherapy. Among these, thymoquinone, the principal bioactive constituent of *Nigella sativa*, has been widely reported to exhibit antioxidant, anti-inflammatory, and anticancer properties in experimental and preclinical models [12,13].

In the present study, we evaluated the effects of TQ, 5-FU, and their combination on the expression of COX-2, IL-6, and IL-8 at both the mRNA and protein levels in two molecularly distinct colorectal cancer cell lines (RKO and SW1116) and in normal human colon epithelial cells (CCD-841CoN). In addition, molecular docking analysis was performed as an independent exploratory approach to investigate potential direct interactions of TQ with the selected proteins. Importantly, the docking analysis was considered separately from the experimental expression data and was not used to infer mechanisms underlying changes in target expression.

Importantly, none of the TQ-monotherapy comparisons versus the untreated control reached statistical significance after correction for multiple comparisons. Although numerical changes were observed for some of the investigated endpoints following TQ treatment, these findings should therefore be regarded as non-significant trends rather than evidence of a statistically significant effect of TQ monotherapy. The only statistically significant reduction in an inflammation-associated mediator observed in the CRC cell lines was the decrease in COX-2 protein levels in SW1116 cells following combined TQ/5-FU treatment.

To our knowledge, this is one of the first studies to integrate gene and protein expression analyses with exploratory molecular docking to investigate responses to TQ, 5-FU, and their combination across COX-2, IL-6, and IL-8 in colorectal cancer cells.

Cyclooxygenase-2 is a well-established mediator of inflammation-associated colorectal carcinogenesis. Unlike the constitutively expressed COX-1 isoform, COX-2 is rapidly induced in response to inflammatory cytokines, growth factors, and other tumor-promoting stimuli, thereby contributing to cell proliferation, angiogenesis, invasion, and metastasis [20]. Increased COX-2 expression has been reported in approximately 85% of colorectal cancers and has been associated with poor clinical prognosis [21]. Consequently, COX-2 has attracted considerable interest as a potential therapeutic target in colorectal cancer.

In the present study, the TQ/5-FU combination significantly reduced COX-2 protein levels in SW1116 cells compared with the untreated control, whereas TQ or 5-FU alone did not produce statistically significant changes. No significant treatment-associated differences in COX-2 protein levels were observed in RKO cells. At the mRNA level, no significant treatment-associated changes in *PTGS2* expression were detected in SW1116 cells. In contrast, in RKO cells, the TQ/5-FU combination significantly increased *PTGS2* mRNA expression compared with the untreated control, despite the absence of a corresponding significant change in COX-2 protein levels. Because mRNA and protein levels were assessed at a single time point, these divergent patterns may reflect differences in the kinetics of transcription, translation, or protein turnover; however, the present experimental design does not allow the underlying regulatory mechanisms to be determined.

Although TQ alone did not significantly reduce COX-2 protein levels in SW1116 cells in the present study, previous studies have reported inhibitory effects of TQ on COX-2 expression in colorectal and other cancer models.

Hsu et al. [14] showed that treatment with 20 μmol/L TQ suppressed COX-2 expression in LoVo colorectal cancer cells and reduced their migratory capacity; these effects were further investigated in a xenograft mouse model [14]. Similarly, Khan et al. demonstrated that TQ downregulated COX-2 expression in lung cancer cells at both the mRNA and protein levels, accompanied by reduced PGE2 production and inhibition of the MAPK and NF-κB signaling pathways [22].

These previous findings provide biological context for the potential modulation of COX-2 by TQ but should not be considered direct confirmation of the treatment response observed in the present study. The differential COX-2 response observed between SW1116 and RKO cells suggests that the effects of the investigated treatments may be cell-line-dependent. Although these cell lines differ in their molecular backgrounds, the present study was not designed to determine which molecular features or signaling pathways account for their differential responses. Therefore, the mechanisms underlying the distinct COX-2 response patterns observed in SW1116 and RKO cells remain to be established.

IL-8 is a multifunctional pro-inflammatory chemokine involved in colorectal carcinogenesis and tumor progression. Elevated IL-8 expression has been reported in several malignancies, including colorectal cancer, where it contributes to cancer cell proliferation, migration, angiogenesis, and metastasis through multiple signaling pathways, including PI3K/AKT signaling [17]. Moreover, increased IL-8 levels have been associated with advanced disease stage and poor clinical prognosis in patients with CRC [23].

In the present study, 5-FU treatment significantly increased *CXCL8* mRNA expression compared with the untreated control in RKO cells. At the protein level, IL-8 levels were significantly higher following 5-FU treatment than following treatment with the TQ/5-FU combination. In CCD-841CoN cells, IL-8 protein levels were significantly higher following combined TQ/5-FU treatment than following TQ treatment alone. These findings indicate treatment- and cell-line-dependent modulation of IL-8 expression and should be interpreted cautiously given the differences between the statistically significant comparisons observed at the transcript and protein levels.

Previous studies have similarly reported that 5-FU can increase IL-8 expression or secretion in colorectal cancer models. Schimek et al. [24] reported increased IL-8 release following 5-FU treatment in colorectal cancer cells, supporting the possibility that *CXCL8*/IL-8 participates in treatment-associated inflammatory responses [24]. Dabkeviciene et al. [25] reported that 5-FU treatment increased *CXCL8* transcript levels by approximately 3.7-fold in parental HCT116 cells and 4-fold in 5-FU-resistant HCT116/FU cells, accompanied by increased IL-8 protein secretion [25]. In addition, Carlsen et al. [26] observed a moderate increase in IL-8 following combined treatment with 5-FU and irinotecan in colorectal cancer cells, irrespective of p53 status [26].

Previous studies have also shown that 5-FU can activate NF-κB signaling and stimulate the expression of pro-inflammatory cytokines, including IL-8, potentially contributing to mechanisms associated with chemoresistance [24,27,28].

To the best of our knowledge, evidence regarding the regulation of IL-8 by TQ in colorectal cancer remains limited. However, Ashour et al. [29] demonstrated that TQ inhibited the growth of hepatocellular carcinoma cells through suppression of NF-κB signaling, accompanied by reduced IL-8 expression and downregulation of IL-8 receptors [29]. Similar observations were subsequently reported in medulloblastoma cells, where TQ inhibited NF-κB activation, decreased IL-8 expression, and induced apoptosis [30]. Collectively, these findings indicate that modulation of IL-8 has been observed as part of the anticancer effects of TQ in different tumor models, although its relevance to colorectal cancer requires further investigation.

In the present study, no statistically significant reduction in IL-8 expression following TQ treatment was observed in the investigated colorectal cancer cell lines. Nevertheless, the differential IL-8 response patterns observed across treatment conditions and cell lines indicate that the regulation of this inflammatory mediator is treatment- and cell-line-dependent. Further studies are required to determine whether TQ directly modulates IL-8-related signaling in colorectal cancer and to elucidate the molecular mechanisms underlying the observed response patterns.

Another important observation of the present study was the limited concordance between mRNA and protein expression of the investigated inflammation-associated mediators. Changes observed at the transcript level were not consistently accompanied by corresponding changes in protein levels. In general, discrepancies between mRNA and protein abundance can arise from differences in the kinetics of transcription, translation, and protein turnover, as well as from post-transcriptional and post-translational regulatory processes [31,32,33]. However, because mRNA and protein levels in the present study were assessed at a single 24 h time point, the mechanisms underlying the observed differences cannot be determined from these data.

A colorectal-cancer-specific example of such regulation is provided by the post-transcriptional control of *CXCL8*. In colon cancer cell lines, the RNA-binding protein HuR has been reported to bind AU-rich elements within the *CXCL8* transcript and increase its stability, thereby prolonging *CXCL8* mRNA half-life and promoting *CXCL8* overexpression [34]. This finding illustrates a potential mechanism by which mRNA stability can influence inflammatory mediator expression. However, HuR activity and *CXCL8* mRNA stability were not assessed in the present study. Moreover, because both mRNA and protein levels were assessed at a single 24 h time point, the mechanisms underlying the observed differences between transcript and protein responses cannot be determined. Therefore, the HuR-mediated regulation of *CXCL8* should be regarded only as biological context rather than an explanation for the present findings. Time-course experiments would be required to characterize the temporal relationship between transcript and protein responses and to investigate the mechanisms underlying their limited concordance.

Data regarding the influence of TQ on IL-6 expression in colorectal cancer remain limited. In SW1116 cells, TQ treatment resulted in a numerical decrease in IL-6 protein levels compared with the untreated control; however, this difference did not reach statistical significance (*p* = 0.07). No corresponding decrease was observed at the mRNA level at the 24 h time point. Given the single-time-point design of the present study and the lack of statistical significance of the protein-level difference, no conclusions regarding the underlying regulatory mechanism can be drawn.

Although direct evidence linking TQ to IL-6 modulation in colorectal cancer is scarce, several studies have demonstrated that TQ targets signaling pathways involved in IL-6 regulation.

Zhang et al. reported that TQ significantly inhibited NF-κB activation in COLO205 and HCT116 colorectal cancer cells, as demonstrated by reduced nuclear phosphorylation of the p65 subunit [11]. Furthermore, Li et al. showed that TQ inhibited both constitutive and IL-6-induced STAT3 phosphorylation in multiple myeloma cells [35]. These findings indicate that NF-κB and STAT3 represent potential pathways through which TQ may influence inflammation-associated signaling. However, because NF-κB and STAT3 activity was not directly assessed in the present study, their involvement in the observed IL-6 response remains hypothetical and requires direct experimental validation.

One of the notable observations of the present study was the different response patterns of the two colorectal cancer cell lines across the investigated treatment conditions. RKO and SW1116 cells differ in their molecular backgrounds, including their MSI/BRAF and MSS/KRAS status, respectively [16]. However, the present study was not designed to determine whether these molecular characteristics contribute to the observed differences in treatment response. Therefore, any association between the molecular background of these cell lines and their differential responsiveness to the investigated treatments remains speculative and requires validation in appropriately designed mechanistic studies.

Although the present study did not investigate the underlying molecular mechanisms, inflammatory signaling pathways may be relevant to the cell-line-dependent responses observed in the present study. NF-κB is a central mediator of inflammation in colorectal cancer and regulates the expression of numerous pro-inflammatory genes, including *PTGS2*, *IL6*, and *CXCL8*. Constitutive activation of this pathway has been associated with tumor progression, resistance to apoptosis, and reduced sensitivity to chemotherapy [11,20,36]. Previous studies have demonstrated that 5-FU can activate NF-κB signaling in colorectal cancer cells, whereas TQ suppresses NF-κB activation [11,28]. However, because NF-κB activity was not directly assessed in the present study, its contribution to the differential responses observed between RKO and SW1116 cells remains speculative and requires experimental validation.

Collectively, the observed response patterns in COX-2, IL-6, and IL-8 varied across treatment conditions and cell lines. Importantly, because none of the TQ-monotherapy comparisons versus the untreated control reached statistical significance after correction for multiple comparisons, the present findings do not provide evidence of a statistically significant independent effect of TQ on these mediators under the experimental conditions used. NF-κB and STAT3 are established regulators of these mediators and may therefore represent potential signaling pathways relevant to the observed response patterns. However, the present study did not directly assess NF-κB or STAT3 pathway activity, including p65, IκBα, or STAT3 phosphorylation. Consequently, no direct mechanistic relationship between the investigated treatments and NF-κB/STAT3 signaling can be established from the present data, and the potential involvement of these pathways should be regarded as a hypothesis requiring direct experimental validation.

Although TQ has been proposed as a chemosensitizing agent in several experimental models, no obvious additive effect of the combined treatment with TQ and 5-FU was observed for the investigated inflammatory mediators under the present experimental conditions. Importantly, a formal quantitative drug-interaction analysis, such as the Chou–Talalay combination index method, was not performed for these endpoints. Therefore, the present data do not allow conclusions to be drawn regarding synergistic or antagonistic interactions between TQ and 5-FU.

From a translational perspective, further investigation of TQ, particularly in combination with conventional anticancer agents, may be of potential interest. Persistent activation of inflammatory signaling contributes not only to colorectal cancer progression but also to therapeutic resistance and tumor recurrence. However, because none of the TQ-monotherapy comparisons versus the untreated control reached statistical significance in the present study, the current findings should not be interpreted as evidence of an independent inflammation-modulating effect of TQ in colorectal cancer cells. Moreover, the present study was designed as a basic in vitro investigation, and the observed effects should not be interpreted as evidence of clinical efficacy. The translational potential of TQ is also limited by its physicochemical and pharmacokinetic properties, including high hydrophobicity, poor aqueous solubility, instability under certain pH conditions, photosensitivity, and low oral bioavailability, which may restrict its absorption, systemic exposure, and delivery to target tissues [37,38,39]. These properties represent important barriers to the clinical translation and druggability of TQ, and nanoformulation-based delivery systems have therefore been investigated to improve its solubility, stability, bioavailability, and tissue distribution [37,38,39]. Nevertheless, further mechanistic, in vivo, and ultimately clinical studies are required to determine the therapeutic relevance of these findings.

As an independent exploratory component of the study, molecular docking analysis was performed to investigate potential interactions of TQ with COX-2, IL-6, and IL-8. Importantly, these computational predictions address a different biological question from the expression analyses performed in the cellular models. Molecular docking evaluates the structural compatibility of a ligand with a protein binding site but does not predict changes in target expression. Therefore, the predicted interactions should not be interpreted as an explanation for the observed changes in mRNA or protein levels. In particular, the predicted interaction between TQ and COX-2 cannot explain the reduction in COX-2 protein abundance observed in SW1116 cells or the absence of a comparable effect in RKO cells. Establishing the functional relevance of a potential direct TQ–COX-2 interaction would require additional experiments assessing COX-2 enzymatic activity or downstream prostaglandin production, such as PGE_2_. Similarly, the predicted interactions with IL-6 and IL-8 do not establish a causal relationship with changes in cytokine expression. Consequently, the docking results should be regarded as exploratory, hypothesis-generating predictions that require independent experimental validation.

Taken together, the present findings demonstrate treatment- and cell-line-dependent response patterns in inflammation-associated mediators. Importantly, none of the TQ-monotherapy comparisons versus the untreated control reached statistical significance after correction for multiple comparisons. The most pronounced statistically significant CRC-relevant reduction observed in the present study, namely the decrease in COX-2 protein levels in SW1116 cells, occurred following combined TQ/5-FU treatment. Therefore, the present data do not provide statistically significant evidence of an independent effect of TQ monotherapy on the investigated inflammation-associated mediators under the experimental conditions used. Further mechanistic, time-course, and in vivo studies are required to determine the biological significance and potential translational relevance of the observed response patterns.

Despite these findings, several limitations of the present study should be acknowledged. First, all analyses were performed at a single experimental time point, which may not fully capture the temporal dynamics of inflammatory mediator expression at the transcript and protein levels. Furthermore, the relatively small number of biological replicates (*n* = 3) limits statistical power and may affect the robustness of the findings. Increasing the number of independent biological replicates would strengthen the statistical robustness of the results and should be considered in future studies. It should also be noted that only one concentration of TQ and 5-FU, selected on the basis of our previous studies, was evaluated; therefore, concentration-dependent effects and interactions between the compounds could not be fully characterized.

An additional limitation of the present study is that IL-6, IL-8, and COX-2 protein measurements were not normalized to viable cell number or total protein measured in the same experimental wells. Previously published MTT data obtained using the same cell lines, treatment concentrations, and exposure period provided parallel information regarding treatment-associated effects on cellular metabolic viability [40]. However, because the MTT assays and protein measurements were performed in separate experiments and different plate formats, these viability data cannot be considered direct normalization factors. Moreover, MTT primarily reflects cellular metabolic activity rather than directly measuring viable cell number. Therefore, the possibility that treatment-associated differences in cell number or metabolic activity contributed to the observed changes in protein levels, particularly for cytokines measured in conditioned medium, cannot be excluded. Future studies should include matched measurements of viable cell number or appropriate measures of cellular biomass together with corresponding normalization of protein measurements. Moreover, the experiments were conducted exclusively in conventional two-dimensional cell culture models, which do not fully recapitulate the complexity of the tumor microenvironment.

Furthermore, the activity of signaling pathways potentially involved in the regulation of the investigated inflammatory mediators, particularly NF-κB and STAT3, was not directly assessed. Therefore, the involvement of these pathways remains hypothetical. Future mechanistic studies should include direct evaluation of pathway activation, including the phosphorylation status of p65 and STAT3 and the regulation of IκBα.

Finally, molecular docking was used as an exploratory, hypothesis-generating approach to predict potential ligand–protein interactions. These computational predictions do not establish direct target engagement or explain the observed changes in target expression and therefore require independent experimental validation. In particular, the functional relevance of the predicted TQ–COX-2 interaction would require confirmation using appropriate functional assays, such as assessment of COX-2 enzymatic activity or PGE_2_ production.

Future studies incorporating multiple time points, broader concentration ranges, three-dimensional culture systems, mechanistic analyses, functional target validation, and in vivo models are warranted to confirm and extend the present findings.

## 4. Materials and Methods

### 4.1. Cell Culture

The human colorectal cancer cell lines RKO (ATCC^®^ CRL-2577) and SW1116 (ATCC^®^ CCL-233), together with the normal human colon epithelial cell line CCD-841CoN (ATCC^®^ CRL-1790), were obtained from the American Type Culture Collection (ATCC, Manassas, VA, USA). RKO and CCD-841CoN cells were cultured in Eagle’s Minimum Essential Medium (EMEM; Sigma-Aldrich, Merck, St. Louis, MO, USA), whereas SW1116 cells were maintained in Dulbecco’s Modified Eagle Medium (DMEM; Sigma-Aldrich, Merck, St. Louis, MO, USA). All culture media were supplemented with 10% fetal bovine serum (FBS; EuroClone, Milan, Italy) and gentamicin (50 μg/mL; BioWhittaker, Lonza, Basel, Switzerland). Cells were maintained at 37 °C in a humidified atmosphere containing 5% CO_2_ using a CO_2_ incubator (Thermo Fisher Scientific, Waltham, MA, USA). Culture media were replaced every 2–3 days, and cells were passaged at approximately 80% confluence using 0.25% trypsin–EDTA (Sigma-Aldrich, Merck, St. Louis, MO, USA). To ensure experimental consistency and preserve cellular characteristics, all experiments were performed using cells within six consecutive passages. Cell morphology and viability were routinely monitored throughout the study. Before each experiment, all cell lines were tested for mycoplasma contamination using the Universal Mycoplasma Detection Kit (ATCC^®^ 30-1012K, American Type Culture Collection, Manassas, VA, USA), according to the manufacturer’s instructions. All cell lines were confirmed to be mycoplasma-free before use in the experiments.

### 4.2. Compound Preparation and Cell Treatment

RKO, SW1116, and CCD-841CoN cells were seeded into six-well plates (Thermo Fisher Scientific, Waltham, MA, USA) at a density of 3 × 10^5^ cells per well and cultured for 48 h to allow cell attachment and stabilization prior to treatment. Cells were then treated with thymoquinone (TQ; Sigma-Aldrich, Merck, St. Louis, MO, USA), 5-fluorouracil (5-FU; Sigma-Aldrich, Merck, St. Louis, MO, USA), or a combination of both compounds. TQ was applied at a final concentration of 20 μM, whereas 5-FU was used at 10 μg/mL (approximately 76.8 μM). In the combination group, both compounds were added simultaneously at the same final concentrations. The selected treatment conditions were based on our previously published dose–response, cell viability, and apoptosis analyses performed using the same cell lines, treatment concentrations, and exposure period [40]. These previous data provided the basis for selecting the experimental concentrations but were obtained in separate experiments and were not used to normalize the protein measurements in the present study.

Stock solutions of TQ and 5-FU were prepared in dimethyl sulfoxide (DMSO; Sigma-Aldrich, Merck, St. Louis, MO, USA). The final DMSO concentration did not exceed 1% (*v*/*v*) in any experimental group. Immediately before use, the working solutions were sterilized by filtration through 0.2 μm syringe filters (Sartorius, Göttingen, Germany). Following 24 h of treatment, both cells and conditioned culture media were collected for subsequent analyses.

### 4.3. Quantitative Real-Time Reverse Transcription PCR (RT-qPCR)

The expression levels of *PTGS2* (encoding COX-2), *IL6* (encoding IL-6), and *CXCL8* (encoding IL-8) were determined by quantitative real-time reverse transcription PCR (RT-qPCR). Relative gene expression was calculated using the 2^−ΔCt^ method, in which the cycle threshold (Ct) values of the target genes were normalized to the geometric mean of two reference genes, glyceraldehyde-3-phosphate dehydrogenase (*GAPDH*) and TATA-box binding protein (*TBP*), to obtain ΔCt values [41]. Normalization to the geometric mean of the two reference genes improved the accuracy and reliability of relative gene expression analysis.

RT-qPCR was performed using the SensiFAST™ SYBR^®^ Hi-ROX One-Step Kit (Bioline, London, UK) according to the manufacturer’s instructions. Amplification reactions were carried out using a LightCycler^®^ 480 Instrument II (Roche Diagnostics, Basel, Switzerland). The amplification program consisted of reverse transcription at 45 °C for 10 min, followed by polymerase activation at 95 °C for 2 min and 45 amplification cycles comprising denaturation at 95 °C for 5 s, annealing at 60 °C for 10 s, and extension at 72 °C for 5 s. Fluorescence signals were acquired at the end of each amplification cycle. Following amplification, melting curve analysis was performed to verify the specificity of the PCR products and confirm the absence of nonspecific amplification and primer–dimer formation. Primer sequences used for amplification of the target and reference genes are listed in Table 3.

All RT-qPCR reactions were performed using three independent biological replicates, with each sample analyzed in triplicate as technical replicates. No-template controls (NTCs) were included in each run to exclude reagent contamination, thereby ensuring the accuracy and reliability of gene expression measurements [42].

### 4.4. Quantification of IL-6 and IL-8 Protein Levels in Cell Culture Supernatants

The concentrations of IL-6 and IL-8 in conditioned cell culture media were determined using the Human IL-6 ProQuantum Immunoassay Kit and the Human IL-8 ProQuantum Immunoassay Kit (Thermo Fisher Scientific, Waltham, MA, USA), according to the manufacturer’s instructions. Both assays are based on ProQuantum™ proximity ligation assay (PLA) technology, which combines antibody-based protein detection with quantitative PCR amplification to enable highly sensitive cytokine quantification.

Briefly, two oligonucleotide-conjugated antibodies bind distinct epitopes on the target cytokine. Simultaneous binding of both antibodies enables ligation of the attached oligonucleotides, followed by amplification of the resulting DNA reporter sequence. The fluorescence signal generated during real-time PCR is proportional to the concentration of the target cytokine in the analyzed sample.

Conditioned cell culture media were diluted 1:2 prior to analysis. Quantitative PCR was performed using the QuantStudio™ 7 Pro Dx Real-Time PCR System (Thermo Fisher Scientific, Waltham, MA, USA), and cytokine concentrations were calculated using the ProQuantum™ Analysis Software v1.0 (Thermo Fisher Scientific, Waltham, MA, USA) according to the manufacturer’s instructions. All measurements were performed using three independent biological replicates. The analytical performance of both assays, including the dynamic range and limits of detection, complied with the manufacturer’s specifications.

### 4.5. Determination of Intracellular COX-2 Protein Levels by ELISA

Intracellular COX-2 protein levels were quantified in cell lysates using the FineTest^®^ Human COX-2 (Cyclooxygenase-2) ELISA Kit (Wuhan Fine Biotech Co., Ltd., Wuhan, China) according to the manufacturer’s instructions. The assay is based on a sandwich enzyme-linked immunosorbent assay (ELISA), in which COX-2 is captured by an immobilized antibody and subsequently detected using a biotinylated detection antibody followed by a horseradish peroxidase (HRP)-conjugated streptavidin complex. The resulting colorimetric signal is proportional to the concentration of COX-2 in the analyzed sample.

Following treatment, adherent cells were washed twice with ice-cold phosphate-buffered saline (PBS) and lysed using the lysis buffer supplied with the kit supplemented with a protease inhibitor cocktail (Sigma-Aldrich, Merck, St. Louis, MO, USA). Cell lysates were incubated on ice for 30 min and clarified by centrifugation at 10,000 rpm for 10 min at 4 °C. The resulting supernatants were collected and used for ELISA analysis.

Standards and samples were analyzed in duplicate. Absorbance was measured at 450 nm using a BioTek Epoch™ Microplate Spectrophotometer (Agilent Technologies, Santa Clara, CA, USA). COX-2 concentrations were calculated from the standard calibration curve generated using the manufacturer’s standards. All measurements were performed using three independent biological replicates.

### 4.6. Molecular Docking Analysis

To investigate the potential molecular interactions of TQ with selected inflammation-associated proteins, molecular docking simulations were performed using COX-2, IL-6, and IL-8 as target proteins. In addition, 5-FU was docked to COX-2 as a computational reference; this comparison was not intended to imply a shared molecular mechanism of action between TQ and 5-FU. For IL-6 and IL-8, blind docking was performed because these proteins lack a comparably well-defined small-molecule binding pocket, allowing potential ligand-binding sites across the protein surfaces to be explored. In contrast, site-directed docking was performed for COX-2 using a defined binding site corresponding to the cyclooxygenase channel, based on the structural characterization described by Rouzer and Marnett [18].

The three-dimensional (3D) structures of TQ and 5-FU were obtained from the PubChem database (https://pubchem.ncbi.nlm.nih.gov/; accessed on 28 June 2026) in Structure Data File (SDF) format and converted to MOL2 format using UCSF ChimeraX version 1.9 (Resource for Biocomputing, Visualization, and Informatics, University of California, San Francisco, CA, USA).

The crystal structures of COX-2 (PDB ID: 5IKR), IL-6 (PDB ID: 1ALU), and IL-8 (PDB ID: 4XDX) were retrieved from the RCSB Protein Data Bank (https://www.rcsb.org/; accessed on 28 June 2026). Protein preparation included the removal of water molecules, co-crystallized ligands, and other non-essential structural components using UCSF ChimeraX version 1.9 (Resource for Biocomputing, Visualization, and Informatics, University of California, San Francisco, CA, USA). The prepared protein structures and ligand files were subsequently processed using AutoDock Tools version 1.5.7 (The Scripps Research Institute, La Jolla, CA, USA) by adding polar hydrogen atoms and assigning Gasteiger partial charges. All receptor and ligand structures were saved in PDBQT format for docking calculations.

Molecular docking simulations were performed using AutoDock Vina version 1.1.2 (The Scripps Research Institute, La Jolla, CA, USA). For each protein–ligand pair, the binding pose with the most favorable (most negative) predicted docking score (kcal/mol) was selected for further analysis. Hydrogen bonds, hydrophobic interactions, and π-interactions were analyzed and visualized using Discovery Studio Visualizer 2025 (Dassault Systèmes BIOVIA, San Diego, CA, USA), UCSF ChimeraX version 1.9, and LigPlot+ version 2.2 (EMBL-EBI, Hinxton, Cambridgeshire, UK).

To validate the molecular docking protocol for COX-2, the co-crystallized ligand ID8 (located in chain A at residue 601 of the COX-2 structure) was extracted and used for redocking validation. The docking search space was defined based on the crystallographic position of ID8. The grid box was centered at X = 38.449, Y = 1.797, and Z = 61.125 Å, with dimensions of 20 × 20 × 20 Å. This search space encompassed the COX-2 cyclooxygenase channel surrounding the co-crystallized ID8 ligand and was subsequently used unchanged for docking of TQ and the reference COX-2 inhibitor celecoxib.

The best-scoring redocked pose was compared with the crystallographic conformation of ID8 using the root-mean-square deviation (RMSD) calculated for corresponding non-hydrogen atoms using PyMOL version 4.6.0 (Schrödinger, LLC, New York, NY, USA). The obtained RMSD value of 1.853 Å indicated good agreement between the crystallographic and predicted binding poses, supporting the ability of the applied docking protocol to reproduce the experimentally observed ligand orientation within the COX-2 binding site.

### 4.7. Statistical Analysis

Statistical analyses were performed using STATISTICA software (version 13.3; TIBCO Software Inc., Palo Alto, CA, USA). All experiments were conducted using three independent biological replicates (*n* = 3 per group). For RT-qPCR analyses, each biological replicate was analyzed in triplicate as technical replicates, whereas ELISA and ProQuantum immunoassays were performed in duplicate according to the respective assay protocols. Technical replicates were averaged within each independent experiment before statistical analysis and were not treated as independent observations.

Because of the small sample size and the limited informativeness of formal normality testing at *n* = 3, non-parametric analyses were used. Differences among experimental groups were assessed using the Kruskal–Wallis test, followed, where appropriate, by multiple comparisons of mean ranks for all groups. Two-sided post-hoc *p*-values were adjusted using the Bonferroni correction within each individual Kruskal–Wallis analysis; no additional global correction across all genes, cell lines, and treatment conditions was applied. Exact adjusted *p*-values are reported in the figures. Statistical significance was defined as *p* < 0.05.

Data visualization was performed using SuperPlotsOfData (University of Amsterdam, Amsterdam, the Netherlands), a web-based application for the transparent visualization and quantitative comparison of continuous data from different conditions [43,44]. The plots show individual biological replicate values together with the group mean and standard deviation.

## 5. Conclusions

The present study identified treatment- and cell-line-dependent response patterns in selected inflammation-associated mediators in colorectal cancer cells. Importantly, none of the TQ-monotherapy comparisons versus the untreated control reached statistical significance after correction for multiple comparisons. Differences between transcript and protein responses were observed across the investigated cell lines; however, because both endpoints were evaluated at a single 24 h time point, the mechanisms underlying these differences cannot be determined from the present study. In SW1116 cells, the TQ/5-FU combination significantly reduced COX-2 protein levels compared with the untreated control, whereas TQ treatment resulted in a numerical, but not statistically significant, decrease in IL-6 protein levels. Thus, the statistically significant reduction in COX-2 protein levels observed in SW1116 cells was associated with combined TQ/5-FU treatment rather than TQ monotherapy. In RKO cells, 5-FU significantly increased *CXCL8* mRNA expression, while the TQ/5-FU combination significantly increased *PTGS2* mRNA expression compared with the untreated control. These findings further emphasize the treatment- and cell-line-dependent nature of the observed responses.

Combined treatment with TQ and 5-FU did not show an obvious additive effect on the investigated inflammation-associated mediators under the present experimental conditions. However, formal quantitative drug-interaction analysis was not performed, and therefore no conclusions regarding synergistic or antagonistic interactions can be drawn. Exploratory molecular docking predicted potential interactions of TQ with COX-2, IL-6, and IL-8, with the most favorable predicted docking score observed for COX-2. These computational results should be interpreted independently of the expression data and regarded as hypothesis-generating observations that require experimental validation of direct target engagement and functional consequences.

Collectively, the present findings do not provide statistically significant evidence of an independent effect of TQ monotherapy on the investigated inflammation-associated mediators under the experimental conditions used. Rather, the results demonstrate treatment- and cell-line-dependent response patterns, including a significant reduction in COX-2 protein levels in SW1116 cells following combined TQ/5-FU treatment. Further mechanistic and time-course studies, together with appropriate in vivo investigations, are required to determine the biological significance and potential translational relevance of these observations.

## Figures and Tables

**Figure 1:**
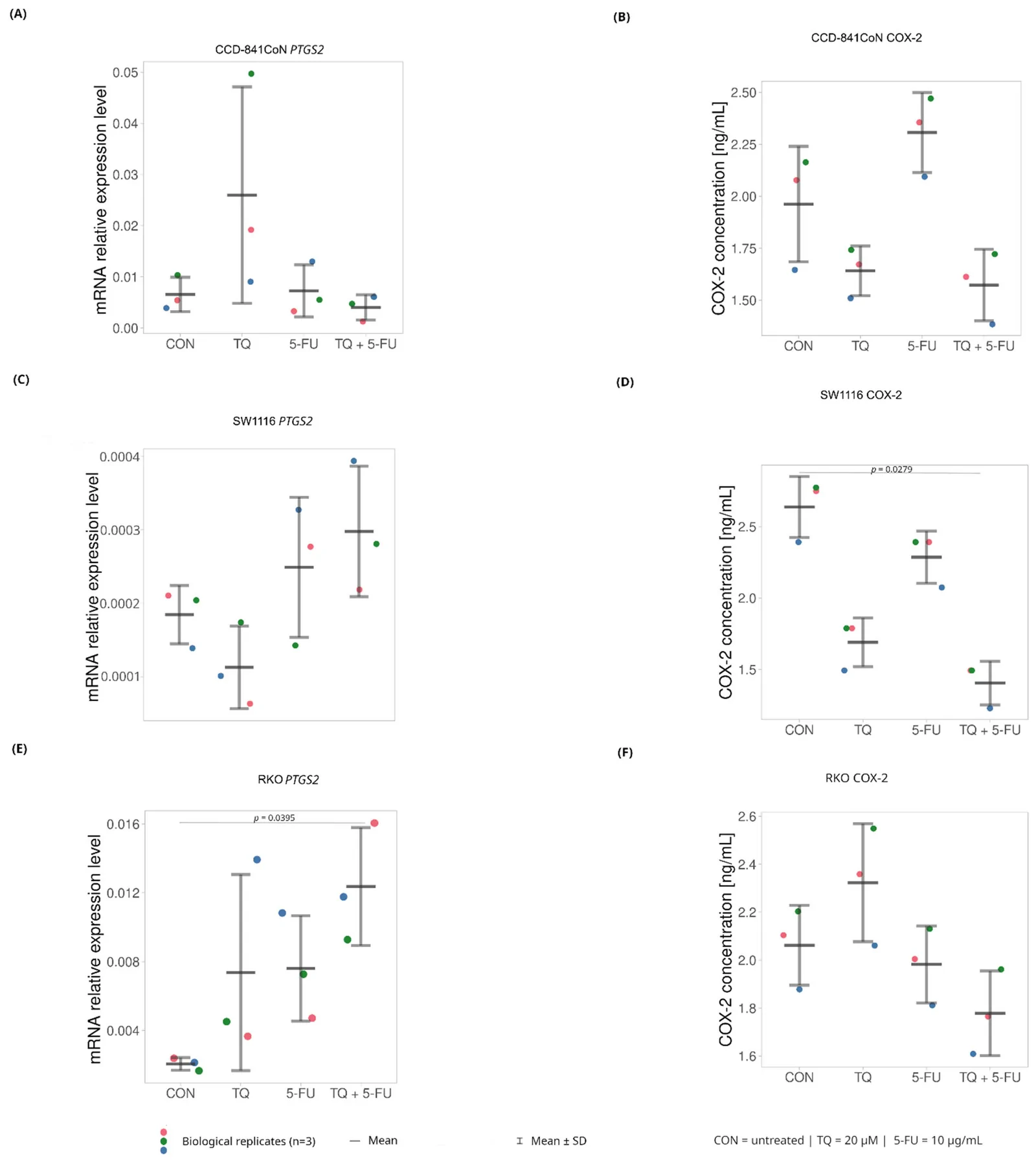
Effects of thymoquinone, 5-fluorouracil, and their combination on COX-2 mRNA and protein expression in colorectal cancer cell lines and normal colon epithelial cells after 24 h of treatment. CCD-841CoN: (**A**) mRNA, (**B**) protein; SW1116: (**C**) mRNA, (**D**) protein; RKO: (**E**) mRNA, (**F**) protein. Colored dots represent individual biological replicates (*n* = 3 per group). The dark-gray horizontal line indicates the group mean, and the gray error bars represent mean ± standard deviation (SD). Statistical significance was assessed using the Kruskal–Wallis test followed by post hoc pairwise mean-rank comparisons with Bonferroni correction. Exact *p*-values are shown above the corresponding comparison brackets. 5-FU, 5-fluorouracil; CON, untreated control; SD, standard deviation; TQ, thymoquinone.

**Figure 2:**
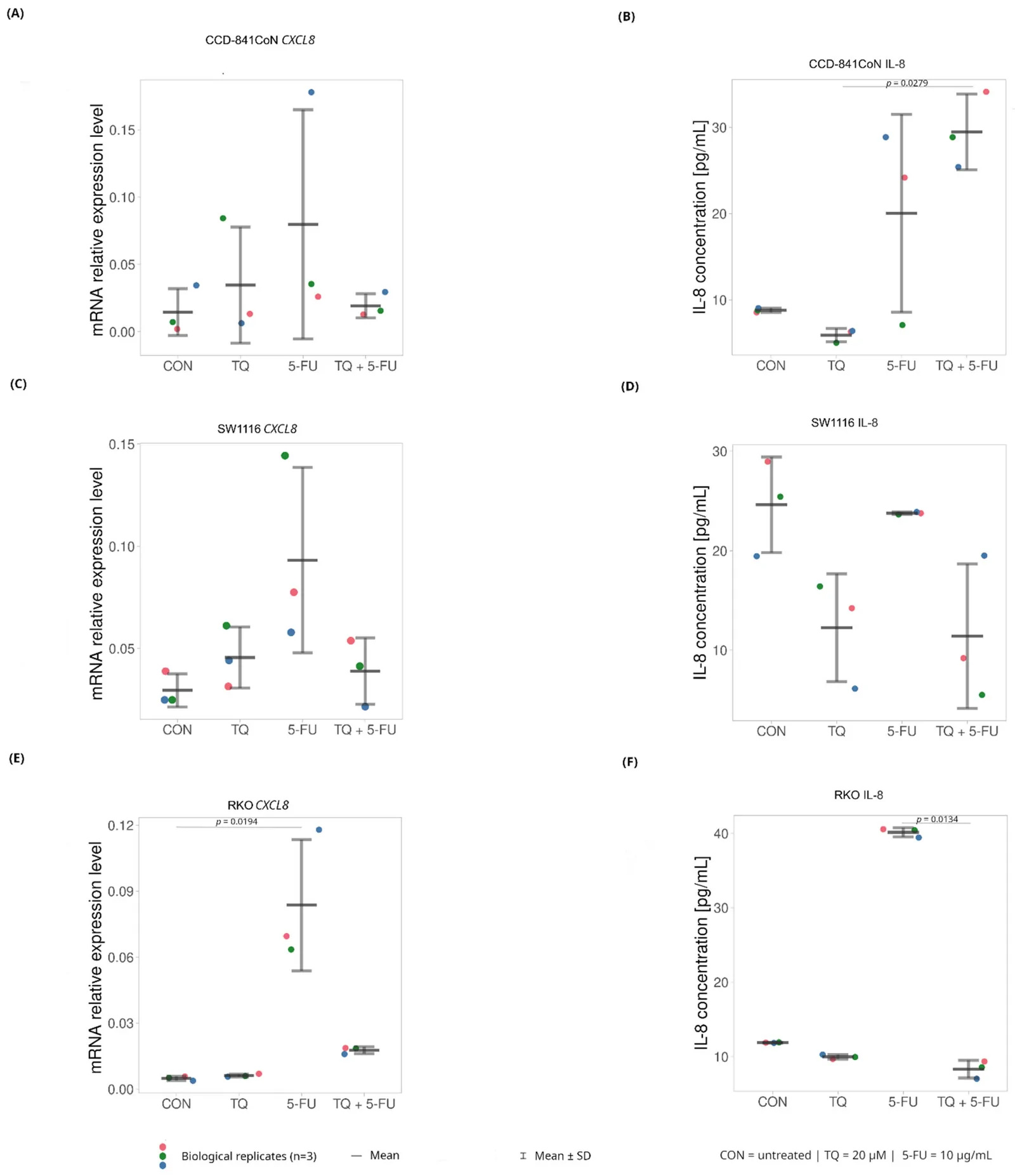
Effects of thymoquinone, 5-fluorouracil, and their combination on IL-8 mRNA and protein expression in colorectal cancer cell lines and normal colon epithelial cells after 24 h of treatment. CCD-841CoN: (**A**) mRNA, (**B**) protein; SW1116: (**C**) mRNA, (**D**) protein; RKO: (**E**) mRNA, (**F**) protein. Colored dots represent individual biological replicates (*n* = 3 per group). The dark-gray horizontal line indicates the group mean, and the gray error bars represent mean ± standard deviation (SD). Statistical significance was assessed using the Kruskal–Wallis test followed by post hoc pairwise mean-rank comparisons with Bonferroni correction. Exact *p*-values are shown above the corresponding comparison brackets. 5-FU, 5-fluorouracil; CON, untreated control; SD, standard deviation; TQ, thymoquinone.

**Figure 3:**
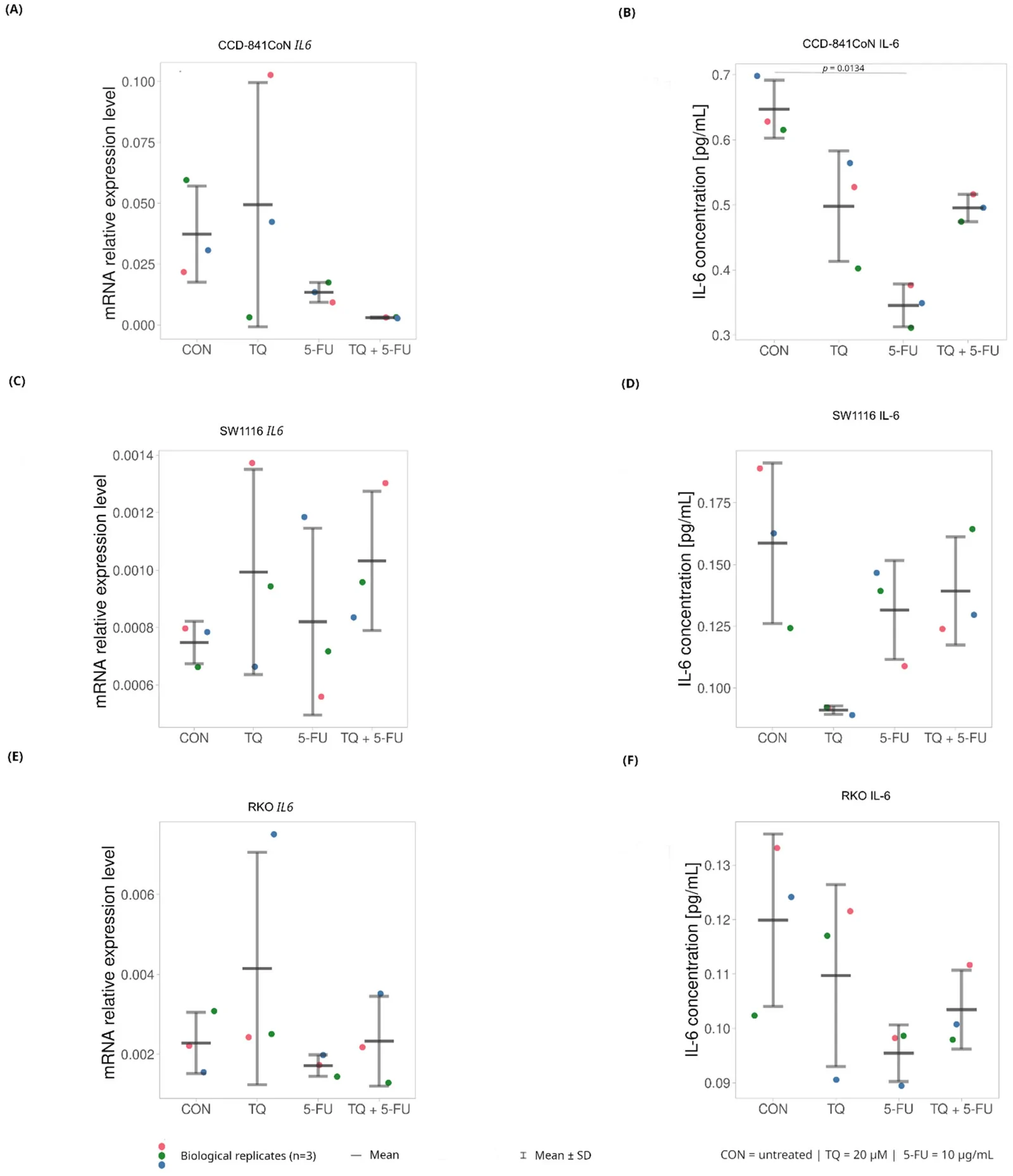
Effects of thymoquinone, 5-fluorouracil, and their combination on IL-6 mRNA and protein expression in colorectal cancer cell lines and normal colon epithelial cells after 24 h of treatment. CCD-841CoN: (**A**) mRNA, (**B**) protein; SW1116: (**C**) mRNA, (**D**) protein; RKO: (**E**) mRNA, (**F**) protein. Colored dots represent individual biological replicates (*n* = 3 per group). The dark-gray horizontal line indicates the group mean, and the gray error bars represent mean ± standard deviation (SD). Statistical significance was assessed using the Kruskal–Wallis test followed by post hoc pairwise mean-rank comparisons with Bonferroni correction. Exact *p*-values are shown above the corresponding comparison brackets. 5-FU, 5-fluorouracil; CON, untreated control; SD, standard deviation; TQ, thymoquinone.

**Figure 4:**
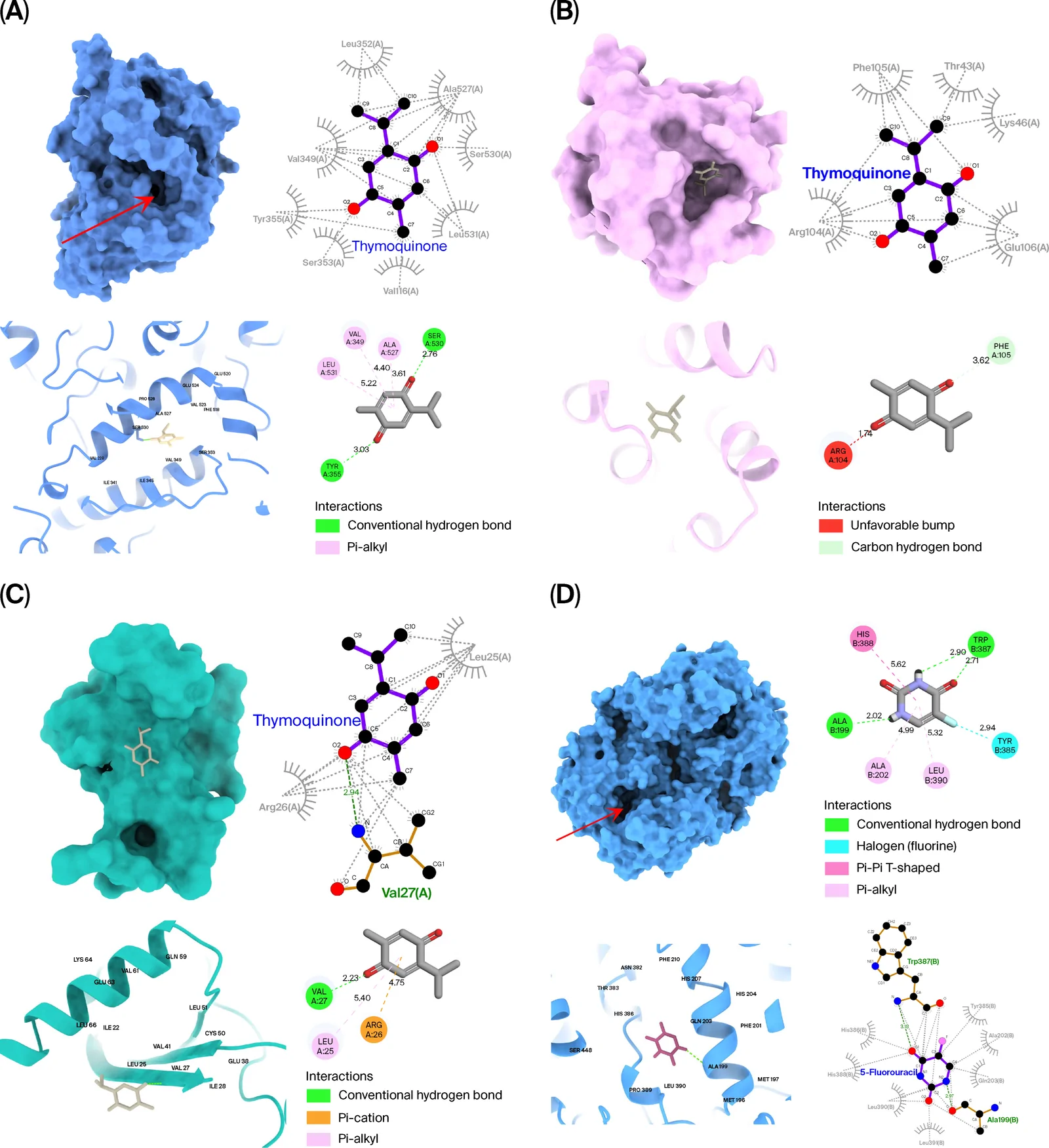
Predicted binding modes and molecular interactions of thymoquinone with COX-2 (**A**), IL-6 (**B**), and IL-8 (**C**), and of 5-fluorouracil with COX-2 (**D**), as determined by molecular docking analysis. Each panel includes the protein surface representation, three-dimensional binding pose, and two-dimensional interaction diagram. Hydrogen bonds are shown in green. The red arrow indicates the predicted ligand-binding site.

**Figure 5:**
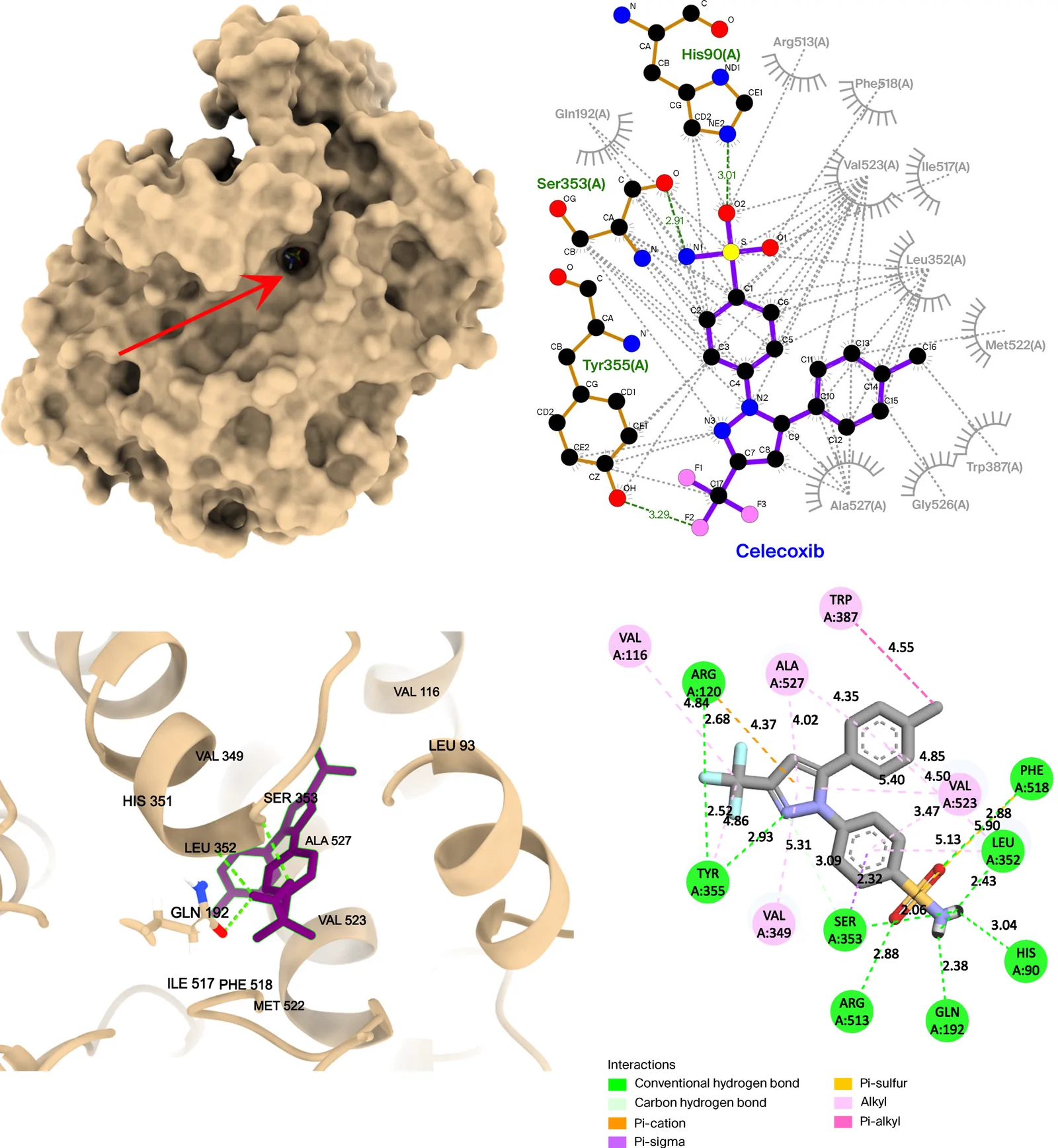
Predicted binding mode and molecular interactions of celecoxib (CXB), used as a reference selective COX-2 inhibitor, within the COX-2 binding site. The figure shows the protein surface representation with the predicted ligand-binding site indicated by a red arrow, the three-dimensional binding pose of celecoxib within the COX-2 binding pocket, and two-dimensional representations of the predicted protein–ligand interactions. Hydrogen bonds are shown in green.

**Table 1:** Comparative summary of molecular docking parameters describing the interactions of thymoquinone (TQ) with COX-2, IL-6, and IL-8.

Docking Parameter	COX-2	IL-6	IL-8
Predicted docking score (kcal/mol)	−6.6	−5.1	−5.2
Hydrogen Bonds	2	0	1
Hydrogen-bonded residues	Ser530; Tyr355	None	Val27
Hydrophobic Interactions	7	5	2
Carbon–hydrogen bonds	0	1	0
Alkyl interactions	1	0	1

**Table 2:** Comparative summary of molecular docking parameters describing the interaction of 5-fluorouracil (5-FU) with COX-2.

Docking Parameter	COX-2
Predicted docking score (kcal/mol)	−5.0
Hydrogen Bonds	2
Hydrogen-bonded residues	Ala199; Trp387
Hydrophobic Interactions	7
Carbon–hydrogen bonds	2
Alkyl interactions	2

**Table 3:** Primer sequences used for amplification of the target and reference genes in RT-qPCR analyses.

Reverse Primer (5′ → 3′)	Forward Primer (5′ → 3′)	Gene Symbol
GAAGATGGTGATGGGATTTC	GAAGGTGAAGGTCGGAGTC	*GAPDH*
GAAGTCCAAGAACTTAGCTG	GCCAAGAGTGAAGAACAG	*TBP*
TGTTGAAAAGTAGTTCTGGG	AAGCAGGCTAATACTGATAGG	*PTGS2*
CTACATTTGCCGAAGAGC	GCAGAAAAAGGCAAAGAATC	*IL6*
GATTGATTTTATCAACAGGCA	CTACATTTGCCGAAGAGC	*CXCL8*

## Data Availability

The original contributions presented in this study are included in the article and its Supplementary Materials. Supplementary Table S1 contains the individual values from the three independent biological replicates for each cell line, treatment condition, and experimental endpoint. Further inquiries can be directed to the corresponding author.

## Supplementary Materials

The following supporting information can be downloaded at: https://www.mdpi.com/article/10.3390/molecules31193415/s1, Table S1. Individual biological-replicate values used for statistical analysis.

## Abbreviations

The following abbreviations are used in this manuscript:

3D	Three-dimensional
5-FU	5-fluorouracil
CIN	Chromosomal instability
COX-2	Cyclooxygenase-2
CRC	Colorectal cancer
Ct	Cycle threshold
CXB	Celecoxib
DMSO	Dimethyl sulfoxide
DMEM	Dulbecco’s Modified Eagle Medium
ELISA	Enzyme-linked immunosorbent assay
EMEM	Eagle’s Minimum Essential Medium
FBS	Fetal bovine serum
GAPDH	Glyceraldehyde-3-phosphate dehydrogenase
HRP	Horseradish peroxidase
IBD	Inflammatory bowel disease
IL-1β	Interleukin-1β
IL-6	Interleukin-6
IL-8	Interleukin-8
IQR	Interquartile range
MSI	Microsatellite instability
MSS	Microsatellite stable
NF-κB	Nuclear factor kappa B
NTCs	No-template control
PBS	Phosphate-buffered saline
PGE2	Prostaglandin E2
PLA	Proximity ligation assay
RMSD	Root-mean-square deviation
RT-qPCR	Quantitative Real-Time PCR
SD	Standard deviation
SDF	Structure Data File
TBP	TATA-box binding protein
TME	Tumor microenvironment
TNF-α	Tumor necrosis factor-α
TQ	Thymoquinone
UC	Ulcerative colitis
