# Supplementary material for: Exploratory Analysis of the Effects of Thymoquinone Alone and in Combination with 5-Fluorouracil on Inflammatory Mediators in Colorectal Cancer Cells: An In Vitro and In Silico Study
Source: Molecules. 2026 Sep 25;31(19):3415. doi: 10.3390/molecules31193415 (PMC13666414; doi:10.3390/molecules31193415)
Supplement: Supplementary file 1 [file molecules-31-03415-s001.zip › molecules-4527518-supplementary.pdf]

Supplementary Materials

Table S1. Individual biological-replicate values used for statistical analysis

Cell line	Target	Endpoint	Treatment	Biological replicate 1	Biological replicate 2	Biological replicate 3	Unit
CCD-841CoN	*PTGS2*	mRNA	CON	0.005407	0.010329	0.003893	Relative expression
CCD-841CoN	*PTGS2*	mRNA	TQ	0.019183	0.008019	0.049687	Relative expression
CCD-841CoN	*PTGS2*	mRNA	5-FU	0.003273	0.005503	0.012984	Relative expression
CCD-841CoN	*PTGS2*	mRNA	TQ/5-FU	0.00127	0.004726	0.006071	Relative expression
SW1116	*PTGS2*	mRNA	CON	0.00021048	0.00020412	0.00013885	Relative expression
SW1116	*PTGS2*	mRNA	TQ	0.00006332	0.00017399	0.00010111	Relative expression
SW1116	*PTGS2*	mRNA	5-FU	0.00027696	0.00014263	0.00032722	Relative expression
SW1116	*PTGS2*	mRNA	TQ/5-FU	0.00021856	0.00028081	0.0003937	Relative expression
RKO	*PTGS2*	mRNA	CON	0.00238	0.001654	0.002133	Relative expression
RKO	*PTGS2*	mRNA	TQ	0.003658	0.004509	0.013926	Relative expression
RKO	*PTGS2*	mRNA	5-FU	0.004713	0.007268	0.010825	Relative expression
RKO	*PTGS2*	mRNA	TQ/5-FU	0.016052	0.009276	0.011763	Relative expression
CCD-841CoN	COX-2	Protein	CON	2.07749	2.16372	1.6455	ng/mL
CCD-841CoN	COX-2	Protein	TQ	1.67205	1.7418	1.50966	ng/mL
CCD-841CoN	COX-2	Protein	5-FU	2.3557	2.47037	2.09409	ng/mL
CCD-841CoN	COX-2	Protein	TQ/5-FU	1.61233	1.72187	1.38426	ng/mL
SW1116	COX-2	Protein	CON	2.74852	2.7727	2.39157	ng/mL
SW1116	COX-2	Protein	TQ	1.78833	1.78833	1.49313	ng/mL
SW1116	COX-2	Protein	5-FU	2.39157	2.39157	2.07418	ng/mL
SW1116	COX-2	Protein	TQ/5-FU	1.49313	1.49313	1.22861	ng/mL
RKO	COX-2	Protein	CON	2.10404	2.20346	1.87811	ng/mL
RKO	COX-2	Protein	TQ	2.35866	2.54888	2.0609	ng/mL
RKO	COX-2	Protein	5-FU	2.00443	2.13058	1.81161	ng/mL
RKO	COX-2	Protein	TQ/5-FU	1.76507	1.96157	1.60901	ng/mL
CCD-841CoN	*CXCL8*	mRNA	CON	0.001987	0.007177	0.034413	Relative expression
CCD-841CoN	*CXCL8*	mRNA	TQ	0.013235	0.084277	0.00625	Relative expression
CCD-841CoN	*CXCL8*	mRNA	5-FU	0.026006	0.035403	0.17794	Relative expression
CCD-841CoN	*CXCL8*	mRNA	TQ/5-FU	0.012797	0.015543	0.029487	Relative expression
SW1116	*CXCL8*	mRNA	CON	0.038904	0.024868	0.024868	Relative expression
SW1116	*CXCL8*	mRNA	TQ	0.031446	0.061196	0.044207	Relative expression
SW1116	*CXCL8*	mRNA	5-FU	0.077538	0.14431	0.057913	Relative expression
SW1116	*CXCL8*	mRNA	TQ/5-FU	0.053846	0.0414	0.021567	Relative expression
RKO	*CXCL8*	mRNA	CON	0.005681	0.005063	0.003739	Relative expression
RKO	*CXCL8*	mRNA	TQ	0.006917	0.005871	0.005582	Relative expression
RKO	*CXCL8*	mRNA	5-FU	0.069507	0.063466	0.11793	Relative expression
RKO	*CXCL8*	mRNA	TQ/5-FU	0.018635	0.018454	0.015877	Relative expression
CCD-841CoN	IL-8	Protein	CON	8.5443	8.77463	9.03856	pg/mL
CCD-841CoN	IL-8	Protein	TQ	6.3095	5.01744	6.39104	pg/mL
CCD-841CoN	IL-8	Protein	5-FU	24.17385	7.07714	28.86372	pg/mL
CCD-841CoN	IL-8	Protein	TQ/5-FU	34.12662	28.86096	25.40247	pg/mL
SW1116	IL-8	Protein	CON	28.94691	25.41492	19.44992	pg/mL
SW1116	IL-8	Protein	TQ	14.22126	16.40979	6.12052	pg/mL
SW1116	IL-8	Protein	5-FU	23.75179	23.62712	23.87646	pg/mL
SW1116	IL-8	Protein	TQ/5-FU	9.18863	5.48794	19.51245	pg/mL
RKO	IL-8	Protein	CON	11.87182	11.91104	11.8326	pg/mL

**Cell line**	**Target**	**Endpoint**	**Treatment**	**Biological replicate 1**	**Biological replicate 2**	**Biological replicate 3**	**Unit**
RKO	IL-8	Protein	TQ	9.68532	9.93617	10.26347	pg/mL
RKO	IL-8	Protein	5-FU	40.56003	40.42205	39.43487	pg/mL
RKO	IL-8	Protein	TQ/5-FU	9.34338	8.56633	7.01745	pg/mL
CCD-841CoN	*IL6*	mRNA	CON	0.021726	0.059537	0.030688	Relative expression
CCD-841CoN	*IL6*	mRNA	TQ	0.10271	0.003145	0.042352	Relative expression
CCD-841CoN	*IL6*	mRNA	5-FU	0.009259	0.017435	0.013454	Relative expression
CCD-841CoN	*IL6*	mRNA	TQ/5-FU	0.003108	0.003207	0.002711	Relative expression
SW1116	*IL6*	mRNA	CON	0.00079689	0.0006624	0.00078435	Relative expression
SW1116	*IL6*	mRNA	TQ	0.001373	0.00094368	0.00066352	Relative expression
SW1116	*IL6*	mRNA	5-FU	0.00055899	0.00071717	0.001184	Relative expression
SW1116	*IL6*	mRNA	TQ/5-FU	0.001303	0.00095811	0.00083554	Relative expression
RKO	*IL6*	mRNA	CON	0.002213	0.003082	0.001553	Relative expression
RKO	*IL6*	mRNA	TQ	0.002427	0.002507	0.007507	Relative expression
RKO	*IL6*	mRNA	5-FU	0.001731	0.001441	0.00198	Relative expression
RKO	*IL6*	mRNA	TQ/5-FU	0.002178	0.001286	0.003518	Relative expression
CCD-841CoN	IL-6	Protein	CON	0.62806	0.61517	0.69801	pg/mL
CCD-841CoN	IL-6	Protein	TQ	0.52735	0.40237	0.56427	pg/mL
CCD-841CoN	IL-6	Protein	5-FU	0.3766	0.31129	0.34918	pg/mL
CCD-841CoN	IL-6	Protein	TQ/5-FU	0.51629	0.47428	0.49561	pg/mL
SW1116	IL-6	Protein	CON	0.18891	0.12427	0.1626	pg/mL
SW1116	IL-6	Protein	TQ	0.091955	0.092131	0.089008	pg/mL
SW1116	IL-6	Protein	5-FU	0.10885	0.13934	0.14668	pg/mL
SW1116	IL-6	Protein	TQ/5-FU	0.12392	0.16432	0.12963	pg/mL
RKO	IL-6	Protein	CON	0.13316	0.10235	0.12415	pg/mL
RKO	IL-6	Protein	TQ	0.12154	0.11703	0.090557	pg/mL
RKO	IL-6	Protein	5-FU	0.098217	0.098625	0.089437	pg/mL
RKO	IL-6	Protein	TQ/5-FU	0.11166	0.097919	0.10075	pg/mL

Values represent three independent biological replicates (n = 3) used for statistical analysis. Where technical replicates were performed, technical replicate values were averaged within each independent biological experiment before statistical analysis. CON, untreated control; TQ, thymoquinone; 5-FU, 5-fluorouracil.
